# Knowledge transmission, peer support, behaviour change and satisfaction in post Natal clubs in Khayelitsha, South Africa: a qualitative study

**DOI:** 10.1186/s12978-020-00957-0

**Published:** 2020-07-08

**Authors:** Hélène Duvivier, Tom Decroo, Aurélie Nelson, Tali Cassidy, Zodwa Mbakaz, Laura Trivino Duran, Virginia de Azevedo, Suhair Solomon, Emilie Venables

**Affiliations:** 1 Médecins Sans Frontières, Brussels, Belgium; 2 Department of Clinical Sciences, Institute of Tropical Medicine, Antwerp, Belgium; 3 Research Foundation Flanders, Brussels, Belgium; 4 Médecins Sans Frontières, Cape Town, South Africa; 5 School of Public Health & Family Medicine, University of Cape Town, Cape Town, South Africa; 6 City of Cape Town Department of Health, Khayelitsha, Cape Town, South Africa; 7 Division of Social and Behavioural Sciences, School of Public Health and Family Medicine, University of Cape Town, Cape Town, South Africa; 8 Southern Africa Medical Unit, Médecins Sans Frontières, Cape Town, South Africa

**Keywords:** HIV, Community participation, Health services accessibility, Postnatal care, Treatment adherence and compliance, social support

## Abstract

**Background:**

The Post Natal Club (PNC) model assures comprehensive care, including HIV and Maternal and Child Health care, for postpartum women living with HIV and their infants during an 18-month postnatal period. The PNC model was launched in 2016 in Town Two Clinic, a primary health care facility in Khayelitsha, South Africa. This qualitative research study aims to understand how participation in PNCs affected knowledge transmission, peer support, behaviour change and satisfaction with the care provided.

**Methods:**

We conducted ten in-depth interviews; three focus group discussions and participant observation with PNC members, health-care workers and key informants selected through purposive sampling. Seventeen PNC members between 21 and 38 years old, three key informants and seven staff working in PNC participated in the research. All participants were female, except for one of the three key informants who was male. Data was collected until saturation. The data analysis was performed in an inductive way and involved an iterative process, using Nvivo11 software.

**Results:**

PNC members acquired knowledge on HIV, ART, adherence, infant feeding, healthy eating habits, follow up tests and treatment for exposed infants. Participants believed that PNC created strong relationships among members and offered an environment conducive to sharing experience and advice. Most interviewees stated that participating in PNC facilitated disclosure of their HIV status, enhanced support network and provided role models. PNC members said that they adapted their behaviour based on advice received in PNCs related to infant feeding, ART adherence, monitoring of symptoms and stimulation of early childhood development. The main benefits were believed to be comprehensive care for mother-infant pairs, time-saving and the peer dynamic. The main challenge from the perspective of key informants was the sustainability of dedicating human resources to PNC.

**Conclusion:**

The PNC model was believed to improve knowledge acquisition, behaviour change and peer support. Participants, staff and the majority of key informants expressed a high level of satisfaction with the PNC model. Sustainability and finding adequate human resources for PNCs remained challenging. Strategies to improve sustainability may include handing over some PNC tasks to members to increase their sense of ownership.

## Plain English summary

There is a need for interventions to prevent HIV transmission from mother-to-child and keep mothers and children healthy after delivery. In 2016, Médecins Sans Frontières, in collaboration with Cape Town City Department of Health and mothers2mothers created Post Natal Clubs (PNC) incorporating mother and child health (MCH) and HIV care in South Africa.

This qualitative research study explores how participation in PNC affected knowledge transmission, peer support, behaviour change and satisfaction for PNC members, staff, and key informants involved in the clubs. The main findings emerged from the data collected through in-depth interviews, focus group discussions and observations. Data was collected until no additional new information was found.

The main benefits of this model were believed to be the combination of care for mother-infant pairs, time-saving and the peer dynamic. The PNC model triggers knowledge transmission on HIV, ART, adherence, infant feeding, healthy eating habits, follow up tests and treatment for exposed infants. Participation in PNC influenced positively health behaviour related to infant feeding, adherence to treatment, monitoring of child health and development, and mothering behaviour. Participation in PNC generated peer support among participants, strengthened relationships and offered an environment conducive to sharing experience. The availability of skilled human resources for the management of PNCs was challenging. Strategies to improve sustainability may include the handing over some tasks to PNC members to increase their sense of ownership.

## Background

Antiretroviral therapy (ART) initiation, adherence, and retention often remain suboptimal among pregnant and postpartum women in low- and middle-income countries. This is due to individual and social-level factors, including poor understanding of HIV, ART and Prevention of Mother-to-Child Transmission (PMTCT), lack of support, fear of disclosure, stigma, poor access to services and health worker attitudes. Poor adherence or retention during pregnancy and the breastfeeding period increase the risk of mother to child HIV transmission and may jeopardise maternal health [1].

In South Africa HIV prevalence is 23.8% amongst women aged 15–49 [2]. In Khayelitsha, a township in the outskirts of Cape Town in the Western Cape Province, with a population estimated to be between 500,000 and one million inhabitants, antenatal HIV prevalence is approximately 30%. Mother to child transmission (MTCT) of HIV is estimated at 0.8% but there is no data on the MTCT rate at 18 months due to poor test uptake, as only 30% of exposed infants return for 18-month testing [2, 3].

The lack of integration of PMTCT and Maternal and Child Health (MCH) services is a major challenge to the successful implementation of PMTCT programs [4]. There is a need for carefully designed and targeted interventions integrating care for mothers living with HIV and their exposed infants during the postnatal period. In order to reduce HIV transmission and to sustain maternal health, it is critical to offer a care model that enhances ART initiation, treatment adherence and retention during pregnancy and the postpartum period. Moreover, even though immunisation coverage in South Africa is relatively high in the first year of life, coverage of other services for mothers and infants is often insufficient [5]. Strategies to improve the utilisation of general postnatal care and postpartum HIV care were adopted by the South African National Department of Health in 2011 [6]. Nevertheless, the implementation of integrated services remains poor [7, 8].

**Fig. 1:**
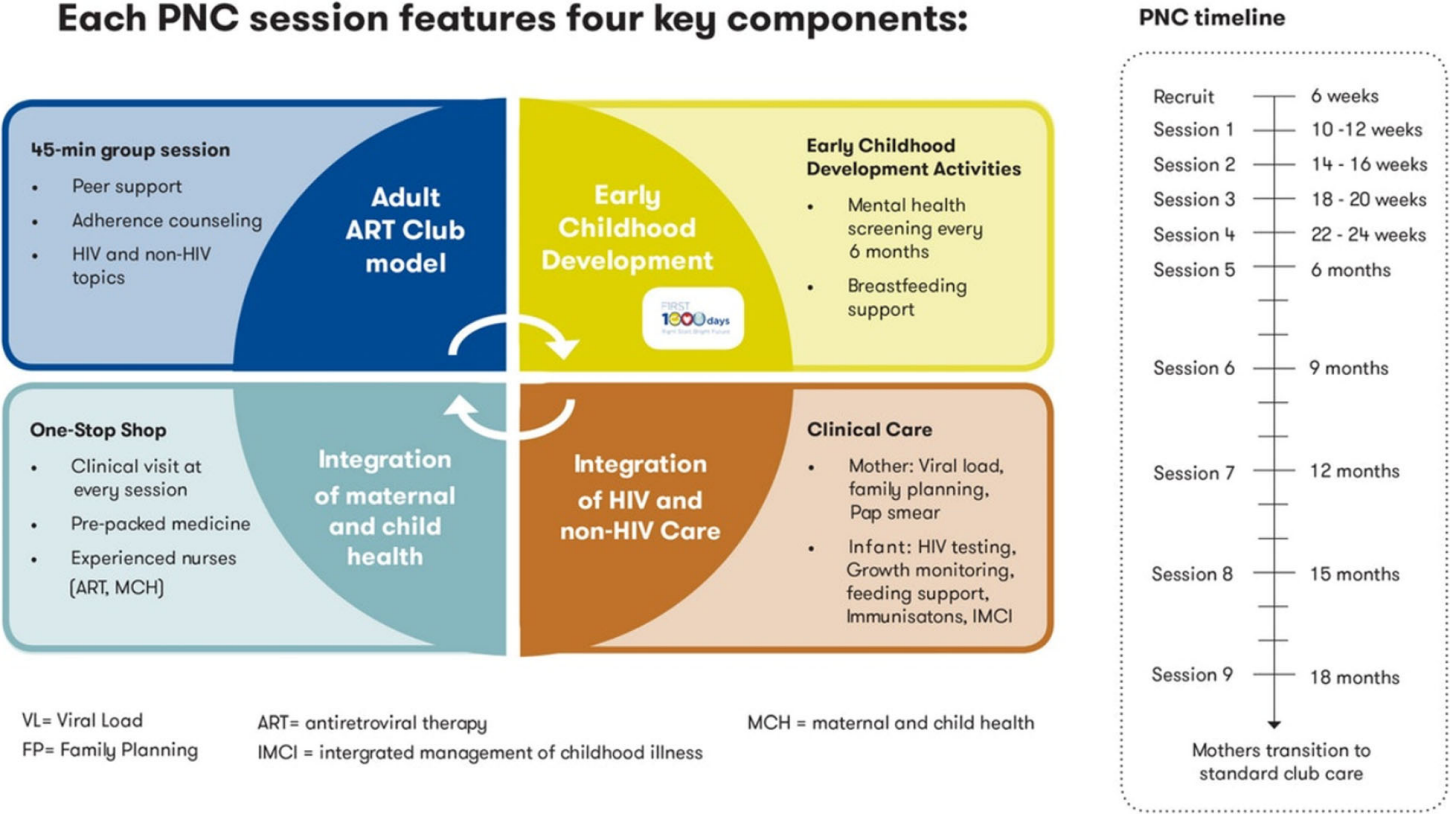
The Post Natal Club model

Peer groups used for counselling and ART refill have shown to be effective in retaining postpartum women in ART care [9]. There is a need to broaden services provided through such models of care to the needs of specific subgroups, such as pregnant and postpartum women. In June 2016, PNCs were piloted in Khayelitsha Town Two clinic by the City of Cape Town Health Department, Médecins Sans Frontières (MSF) and mothers2mothers (m2m). The latter is an Africa-based, global non-governmental organisation empowering mothers living with HIV to support other mothers, their families and their communities. The intervention offers a comprehensive package of care to postpartum women living with HIV and their infant(s). The PNC model (Fig. 1) integrates PMTCT and general MCH care during the postnatal period. It includes mental health and educational components and is delivered to postpartum women and their infants during an 18-month postnatal period. Facility and community-based m2m mentor mothers provide peer’s support and health education on PMTCT and Early Childhood Development (ECD).

This qualitative study was conducted at Town Two clinic in Khayelitsha, in the Western Cape Province, of South Africa. It is the first study that explores how participation in PNC affected knowledge transmission, peer support, health seeking behaviour and satisfaction with the care provided.

## Methods

### Study design

This was a qualitative study consisting of focus group discussions, interviews and participant observation conducted between October 2017 and November 2017.

### Study setting

The PNC model was piloted at Town Two Clinic, a primary health-care clinic in Khayelitsha, by the City of Cape Town Department of Health in collaboration with MSF and m2m. In 2016, 194 infants of HIV infected women attended postnatal PMTCT services at Town Two. From June 2016 onwards, postpartum women living with HIV seeking care at the clinic were invited to join a PNC.

### Sampling and recruitment

Purposive sampling was used to select 1) seventeen postpartum women living with HIV enrolled in a PNC at Town 2 clinic for at least 6 months; 2) three key informants from the City of Cape Town Department of Health involved in the design and implementation of PNCs and 3) seven staff involved in the facilitation of PNCs (MSF nurses and counsellors and m2m mentor mothers).

The counsellor working in the PNC contacted PNC members by phone or in person at the clinic (in case they did not have a phone or did not respond when the counsellor called) to invite them to participate in the study. Those who expressed an interest in receiving further information about the study were referred to the research team and were given the choice between taking part in an in-depth interview (IDI) or a focus group discussion (FGD).

Key informants from the City of Cape Town Department of Health and PNC staff were identified by the programme manager of the PNC and contacted telephonically about the study. Those who were interested in participating were then referred to the principal investigator (PI), who provided more information on the study. Three key informants were identified by the programme manager of MSF. All agreed to participate. Seven staff involved in PNCs at the time of the study also agreed to participate in the study.

Club members were informed in advance that researchers would be present during their next session to conduct participant observation. Information about the study was provided by the researchers prior to the start of the session observed and verbal consent was obtained from all members that were present before the observations began.

### Reflexivity

Data were collected by the PI and the research assistant. The PI is a Belgian woman with a Master’s in Public Health who had previously worked for MSF, including implementing community models of care, and is trained in qualitative research methods. Her affinity with the topic could have created an interviewer bias in favour of community participation, but this was mitigated by working with a local research assistant and discussing findings with the research team during data collection and analysis. The research assistant was a Xhosa-speaking, South African woman who has worked as counsellor and had previous qualitative research experience. The PI and the research assistant did not know any of the participants before the start of the study and were not directly working with the PNCs at the time of data collection.

### Data collection tools

Guides using open-ended questions were developed for the different subgroups participating in IDIs and FGDs. The guides were developed in English, then translated into isiXhosa and pre-tested to ensure participants’ understanding.

### Data collection

Ten IDIs and three FGDs were conducted in isiXhosa and/or English. In addition, the PI and the research assistant carried out participant observation during two routine PNC meetings to observe education sessions, counselling, peer support and mother-infant interactions (Table 1).

**Table 1:** Study participants per data collection methods

	Data collection methods		
	Focus group discussions (***N*** = 3)	In-depth interview (***N*** = 10)	Participant observation (***N*** = 2)
**PNC staff**	One focus group discussion with seven participants (in English)	_	Two PNC sessions observed with four staff participants (in isiXhosa)
**Key informants**	_	Three participants (in English)	_
**PNC members**	Two focus group discussions with five participants each = 10 participants (in isiXhosa)	Seven participants (Six in isiXhosa and one in English)	Two PNC sessions observed with five PNC participants (in isiXhosa)
**Total participants per data collection method**	17	10	9

IDIs with PNC members and the FGDs with PNC staff were carried out in the clinic. The FGDs with PNC members took place in the MSF office in Khayelitsha. Participant observation took place during PNC meetings at the clinic.

The mean duration of IDIs was 47 min, and the mean duration of FGDs was 65 min. Participant observation lasted approximately 2 hours, the length of the PNC sessions.

All IDIs and FGDs were audio-recorded.

The PI had daily meetings with the research assistant during the data collection period to conduct intermediate analysis, adapt the interview guides, and assess saturation [10].

### Transcription, coding and data analysis

Audio-recordings were transcribed verbatim and notes taken during participant observation were summarised in English immediately after the sessions. Data collected in isiXhosa was translated simultaneously into English during the transcription process by the research assistant after each IDI or FGD.

All IDI and FGD transcripts and summaries of participant observation were coded by the PI using NVivo qualitative data analysis software (version 11). Codes and sub-codes were generated inductively based on concepts emerging from the data. Codes emerging from the first IDIs, FGDs and participant observation helped to create a coding tree, which was adapted along the data collection process. Thematic content analysis was used to analyse the data.

The four principal themes of the coding tree were1) peer support generated by PNCs, 2) knowledge acquired in PNCs, 3) behaviour change triggered by PNCs and 4) satisfaction with the PNC model. Sub-themes were identified under each theme.

Findings were validated through the triangulation of data collected from different data collection techniques (IDIs, FGDs and participant observation) and different type of study participants (patients, staff, key informants) to develop a broader and deeper understanding of the data.

## Results

We present the views of PNC participants, staff and key informants on the knowledge acquired through PNCs and their influence on behaviour change. We then explore peer support and behaviours generated by the PNCs and the satisfaction with the care provided.

All interviewees apart from one were female. All PNC members who were interviewed were female and between 21 and 38 years old. Detailed demographic information about PNC staff and key informants has not been divulged as it could make them easily identifiable.

### Knowledge acquired in PNCs

#### Knowledge on HIV, ART and adherence

Participants in PNCs reported acquiring knowledge about HIV, CD4 count, viral load and PMTCT during the PNC sessions.

“The *thing we are asked when we arrive is how we are keeping up with the pills. You are asked the name of the pills you are taking. I was never asked that before*” (PNC member, FGD, 25 years old).

They mentioned learning about their treatment and various adherence strategies such as setting treatment reminders or knowing what to do in case they missed a dose.

*“I didn’t know the trick of taking pills and separating them. You put them in different bags and other places. It was explained at PNC [...] You know that when you lose your bag, you have [ARVs] in another bag and that when you return [home], you return alright and healthy” (*PNC member, FGD, 31 years old).

#### Knowledge on feeding

Mothers who experienced challenges with breastfeeding their infants said that they felt supported and reassured by the m2m mentor’s advice. Some mothers mentioned that they felt “*free to breastfeed because of their education*” (PNC member, FGD, 21 years old). PNC participants reported gaining information on the importance of exclusive breastfeeding until 6 months.

*“I was educated about the importance of breastfeeding a baby. I must feed him breast milk only and not mix feed”* (PNC member, FGD, 21 years old).

“*When I got to hear from the clinic that he [the baby] was not supposed to eat [solid food] before six months because he may be infected with HIV, I told my granny because I’m open about my status. I told her that the baby is not supposed to eat anything but the milk”* (PNC member, IDI, 21 years old).

Mothers who decided not to breastfeed their child received information on bottle feeding. Some participants also reported that PNCs helped them to correct common misperceptions and beliefs about infant feeding. Mothers said that they learned tricks to encourage their infants to eat solid food after 6 months and reported that PNC participation increased their awareness about the importance of a healthy diet and good hygiene.

“*If I am preparing a bottle, I must boil water correctly and pour it in a flask. If I am preparing food, if it’s a lot, I must store it in Tupperware and check the dates, put it in the fridge and warm it up before using it” (*PNC member, IDI, 25 years old)

#### Knowledge of infants’ follow-up tests and treatment

During PNC sessions, the group *“would talk about the test that is going to be done on the baby”* (PNC member, FGD, 27 years old). Mothers described being prepared about the possible results through pre-test counselling provided in PNCs.

“*They [the mothers] have already decreased their fears because they’ve spoken about it [HIV testing] in the previous session”* (PNC staff, FGD2).

PNC participants stated that they acquired knowledge on how to administer prophylaxis to their infant during the breastfeeding period.

*“From zero to one month, a child will receive Nevirapine. If you decide to breastfeed the baby, you will receive Nevirapine syrup. He will drink that syrup until stopping breastfeeding”* (PNC member, FGD, 23 years old).

*“I mustn’t give the infant medication that is not prescribed by a nurse or the doctor”* (PNC member, FGD, 31 years old).

One PNC member reported acquiring knowledge on the importance of communicating with household members about their treatment: *“They must know this medicine works to stop him from getting HIV”* (PNC member, FGD, 25 years old).

#### Knowledge on early childhood development activities

Participants reported that they learned how to hold and play with their infant so that he or she can grow. They mentioned learning exercises and playful activities such as crawling:.

“*They teach us there how to handle a baby … so that he can grow. You put him on his stomach. He must crawl. There, you teach him exercises.”* (PNC member, FGD, 25 years old).

PNC staff explained that they taught songs to the mothers to encourage them to play with their babies.

“*We’re also singing and showing them the body parts, we sing head and shoulders, chest and waist … It helps them a lot because they can learn. The babies can learn that this is my head because when you are singing that song that “head and shoulders”, you’re sitting with your baby in front of you, showing the parts that you are talking about” (*PNC staff member, FGD).

### Peer support generated by post Natal clubs

The relationship between PNC members was reported to grow over time. Some PNC members pointed out that the PNCs made them feel *“equal”*, which helped them integrate in the group.

*“They [mentor mothers] create an environment that is conducive for everybody to feel comfortable because they themselves are HIV positive and they share their own stories”* (PNC staff, FGD).

#### Relationships

PNC staff reported that PNC members formed relationships that went beyond their meetings at the clinic. Mothers reported communicating through WhatsApp messages, meeting in each other’s houses or in the community and supporting each other with material things such as infant clothes or babysitting.

“*They [PNC participants] built that friendship and they share more than we think they are sharing because they even share their own social problems on WhatsApp groups they created … The Post Natal Clubs give them a platform where they can form better relationships and better sisterhood”* (PNC staff, FGD).

“*We assist each other mostly with baby things. If one of us has a shortage of baby things, then the other one would provide something like food and other things. If maybe if your baby had outgrown their clothes, there are some things you would give to the one in need. Even if one of us has to go to look for a job, you would ask one of the available moms in the group to keep your baby while you go and look for work” (*PNC member, IDI, 31 years old).

“*My child was the first one to be born in our group of children; we made a [birthday] party and invited each other. Then there are those who were born in August. We invited each other again because our children are in the same age group” (*PNC member, IDI, 25 years old).

#### Knowledge transmission to community members

PNC members shared the information they received in education sessions with family members and friends. A mother reported that when she goes to the PNC session, some community members say: *“You have to go there and bring back the news to us and advise us”* (PNC participant, FGD, 38 years old).

PNC members are also believed to provide counselling and information learned in the PNC to others in the community.

*“That information that they’ve got there, it’s not like they’re going to keep it for themselves. They’re going to share it with somebody else”* (Key informant from the City of Cape Town Department of Health)

#### Conflict of interest

PNC members and staff stated that PNC staff support club members in disclosing their HIV status, and most participants stated that they had disclosed to partners or family members.

“*When I attended this Town Two Club, I did not want to tell people outside but when I met mothers here at Town Two, I was able to disclose. I do not have problems, even now I am alright”* (PNC member, FGD, 34 years old).

“*When I first came, I didn’t disclose to my husband but after participating in the Club, I was encouraged to disclose, and I ended up disclosing to my husband. Before, it caused difficulties when taking my treatment because I used to check where he was and then I would take the treatment in his absence”* (PNC member, IDI, 36 years old).

*“It [PNC] has affected me in such way that I was able to disclose my status at home”* (PNC participant, 31 years old, IDI 8).

One key informant explained that sharing information about how they disclosed amongst peers in the PNC is more beneficial than clinicians telling them that they should disclose.

#### Advice and role models

PNC members explained how they advised each other as parents*.* They told each other to *“take the infant to the clinic”* (PNC member, IDI, 31 years old) in case of worrying symptoms. They also reported advising each other on personal issues, adherence and disclosure.

“*Advice would be around disclosure. She* [a PNC member] *might have difficulty taking treatment in front of other people in the house. So, they* [other PNC members] *would advise her … They would say she needs to disclose when she is ready. But she needs to take her treatment” (*PNC member, IDI, 36 years old).

*“When I was pregnant, my baby’s father denied the baby was his. He came back but he didn’t contribute towards taking care of the baby. So, I was affected by that … The fellow group members advised me when I shared this to them, telling me: “It happens”, I mustn’t stress myself out about it because it’s going to affect the way I’m taking my medication and might affect the baby” (*PNC member, IDI, 36 years old).

They reported supporting each other’s adherence when meeting outside the clinic setting. At parties, they would ask each other *“Hey friend, did you take your things [ART]? Put your alcohol here. You should have already taken your pill and then you can enjoy yourself”* (PNC member, IDI, 25 years old).

#### Stigma reduction

Mothers described how PNCs helped them to deal with external and internal stigma related to HIV. They mentioned that they disclosed their status to some family members or their partner, which often generated a feeling of relief and could trigger support from family members. One PNC staff member stated that the clubs had *“really improved their lifestyle”*.

“*Some of them even cried saying: ‘Before I was so afraid of HIV and I was very shy. I couldn’t talk about it. I couldn’t come to terms with it but since I started with the Post Natal Clubs, I accepted it. I know how to live with it positively and I’m very happy. I wish you can take this to other people as well’. We’re getting positive feedback from the mothers” (*PNC staff, FGD).

“S*tigma could end or be reduced … Now, we are able to stand up and talk”* (PNC participant, IDI, 25 years old).

*“When I arrived here [in PNC], I realised that I cannot blame myself because at the end I am already like this. I must accept myself in order for my child to live happily”* (PNC participant, FGD3).

### Influence of post Natal Club participation on behaviour

PNC participants described how they adapted their behaviour in relation to health, breastfeeding and adherence based on the advice received in PNCs.

#### Health related behaviour

Many mothers reported that they took the decision to breastfeed their child as a result of the education on breastfeeding provided in the PNC. They learnt to breastfeed exclusively until the infant reached 6 months old and then started to give them solid food. Some mothers also said that PNCs influenced them to monitor their health and that of their infants.

*“In my view, PNC helped me to monitor my health and my baby’s health and not do things the way I used to and be ignorant about my health.”* (PNC participant, 25 years old, IDI5).

“*I learned more in PNC club about breastfeeding. My child didn’t have diarrhoea … My baby was not sick. I made sure to feed my baby the way they said it: ‘Don’t mix feed’, ‘don’t give water’, ‘don’t give anything’. At home, they are amazed: ‘This clinic of yours, the child does not get sick. It is the first time we see a baby who doesn’t have diarrhoea”* (PNC member in FGD1, woman, 27 years old).

According to PNC members, PNCs were also believed to modify behaviours related to ART adherence.

“*I was able to disclose my status at home and I would ask someone to go fetch my medication if I was going to be absent, and would also take my baby to the clinic” (*PNC member, IDI, 31 years old).

“*I do not want to lie. I was a person who was not taking treatment … When I joined this group, I started to take my treatment. I would even ask my baby’s dad to remind me because I do not want my kids to be looked after by another person. I want to stay with them, so he also encouraged me to take my treatment … They encouraged me a lot at the club”* (PNC member in FGD3, woman, 29 years old).

PNC members also said that the education provided by the mentor mothers motivated them to use condoms with their partners.

#### Motherhood

PNC members said that PNCs helped them to gain confidence about their ability to breastfeed and care for their infant. Some mothers explained that mentor mothers helped them to *“put the infant in the right position and enjoy [breastfeeding]”* (PNC participant, FGD). Mothers said they learned to sing songs and make toys such as mobiles to hang above the bed or shakers (maracas) for their infants in PNCs. They played with their children and learned to monitor early childhood development.

*“Now, I know that a baby can hear. So, I must speak to my baby and I must play with him.”* (PNC participant, IDI, 21 years old).

“*During the session we give the mothers ideas for how they can make their babies toys to play. We make mobiles, we make shakers, we also sing.”* (PNC staff in FGD2).

### Satisfaction with the PNC model

PNC advantages highlighted by PNC members, staff and key informants included the following:

#### Sharing experiences

Study participants perceived PNC as an opportunity for members to share about their experiences among themselves and with mentor mothers who went through similar situations. They felt they could receive the appropriate support by peers and felt understood.

*“There, you can reveal what is eating you inside and share it freely with others”* (PNC member, IDI, 31 years old)

#### Satisfaction with the care provided and staff attitude

PNC members, staff and key informants expressed high level of satisfaction with the quality of the care in PNC.

*“We are taken care of, with my child at the same time, and the care they give is excellent”* (PNC participant, FGD, 27 years old).

They also appreciated the comprehensive aspect of the care provided to the mother-infant pair. Believing PNCs to increase efficiency and save them time:

*“Everything is being done in one place by one clinician”* (Key Informant, IDI).

They found that the staff treated them with respect.

*“We’re getting the respect we want”* (PNC member, IDI, 21 years old).

#### Satisfaction with the outcomes generated by PNCs

Study participants reported that PNC increased health education on PMTCT related topics.

*“You come to club with no information and you come out of it with information that will help you”* (PNC member, IDI, 36 years old).

They also found that the PNC model enhanced appropriate clinical follow up, including mental health screening and referral for further care.

PNC members also expressed a high level of satisfaction regarding the support members are providing to each other outside of the clinic with material assistance.

### Challenges related to PNCs

Some PNC members complained that some sessions involving particular medical procedures, such as drawing blood for viral load testing, created lengthy waiting times*.* Others mentioned issues relating to confidentiality as beneficiaries attending other clinical consultation could walk through the room where PNCs take place.

*“If you have not disclosed at that time [ …*] *a person who knows you would already know about your status* [if they see you attending the PNC]*”* (PNC participant, 28 years old, IDI3).

According to key informants involved in PNC implementation, challenges included the need to allocate space for PNCs and issues relating to workload.

*“The space is a big challenge [ …*] *We don’t have enough space [ …*] *Sometimes we have to change the time for other clubs because now it clashes with the Post Natal Clubs”* (Key informant, IDI).

*“The administration for each patient is probably as long as the consultation”* (Key informant, IDI).

Some staff expressed that the group model may not be convenient for mothers who are afraid to disclose their status.

*“We inform them when we recruit them that [ …*] *there will be other mothers. Some of them might not be ready to disclose but they are not forced into the clubs”* (PNC staff, FGD).

One key informant expressed doubts about the sustainability of the model because, at the time of the study, it was heavily supported by m2m and MSF.

“*If you’d used clinic staff, it would not have been the same because clinic staff cannot sit for eight hours doing 10 mothers and infant pairs there. The workload of those professional nurses has in fact plummeted”* (Key Informant, IDI).

## Discussion

This study is the first to show how a comprehensive package of care provided to a peer group of postpartum women living with HIV and their infant(s), the PNC model, triggers knowledge acquisition, behaviour change, peer support and satisfaction from the perspective of PNC participants, staff and key informants. Most mothers said that PNCs helped them to learn important information about their child health and their own which influenced positively their motherhood and health seeking behaviours. PNCs seem to trigger peer support and generate high satisfaction with the care provided. PNCs effected participants’ behaviour by improving adherence to treatment, feeding habits, and early childhood development stimulation. Indeed, education and peer support are well known enablers of adherence and retention in ART care [11, 12].

In addition to the benefits for PNC participants, staff and key informants highlighted the positive impact of PNCs on having updated data on PNC participants’ health outcomes. Quantitative data collected in PNCs from July 2016–June 2018 showed that among 335 mothers and 340 infants recruited in PNCs, uptake of infant HIV testing was 96.8% at 9 months of age and 94.7% at 18 months of age and no infant seroconversions had occurred. Maternal viral load testing and suppression remained above 90%**.** Regarding vaccination coverage, of 187 infants who had been 12 months in PNC, 96.2% were fully immunised [3].

Another study confirmed that the integration of PMTCT and MCH services during the postnatal phase improves MCH outcomes. A randomised control trial conducted in South Africa showed that 77% of women who received integrated postnatal care together with their infants achieved viral suppression at 12 months postpartum, compared to 56% in the group of women receiving standard ART and MCH services separately. The same study also showed that breastfeeding and exclusive breastfeeding lasted longer in the group receiving integrated MCH and PMTCT care versus the control group (6.9 versus 3.0 months, *p* = 0.006, and 3.0 versus 1.4 months, *p* < 0.001, respectively) [13].

The main criticism regarding the model came from key informants who questioned its sustainability and thought that the model increased the workload for staff involved. Key informants reported that the expansion of the PNC model could be jeopardised by human resource constraints and a strong dependency on MSF and m2m support. Indeed, previous research showed that the sustainability of projects offering care to specific target groups depends on the successful training of people at various levels, including non-medical staff, and the active participation of community members [14]. We recommend, therefore, increasing the level of involvement of graduated PNC members in their own follow-up and in providing support to their peers. The more that patients become autonomous for routine tasks related to their chronic care, the more sustainable approaches promoting patient participation become [15]. A study conducted in Kenya showed that ART and adherence support by People Living With HIV (PLWHIV) resulted in no difference in virologic, immunologic or clinical outcomes in patients compared with a control group receiving standard ART dispensation and adherence support at the clinic. Community-based care by PLWHIV resulted in similar clinical outcomes as usual care but with half the number of clinic visits. This pilot study suggests that task-shifting can result in safe and effective community-based care for PLWHIV [16]. A study conducted in Tete, a rural area in Mozambique, on a community model of care called the Community ART Groups (CAGs) also suggests that patients can play a more active role in activities, such as counselling, drug dispensation and administrative tasks, which increased sustainability [17, 18].

To enhance the sustainability of the PNC model, we recommend evaluating if PNCs can function more autonomously, particularly in relation to administrative and medical routine tasks and peer education. Future studies could evaluate if peer-support generated between PNC members could aid the formation of postnatal CAGs or Community Adherence Clubs for mothers to continue supporting each other after graduation and to assist them to access treatment in the community [19]. It would be important to assess the support provided by expert graduates to mothers who recently joined a PNC. Former PNC participants could have a larger degree of involvement and take a leading role in providing health education, dispensing pre-packed treatment, sharing information among participants and promoting PNCs in the community.

Our study has several strengths and limitations. Our findings were validated through methodological and data triangulation. Data collection was continued until intermediate analysis showed saturation. One limitation of this study is that data collection did not involve the mothers who refused to join PNCs, or PNC participants who had dropped out of the clubs. These women could have taught us more about the barriers to joining and staying in PNCs as well as the reasons why women chose to leave. Another limitation was related to the language in which data collection took place. Most interviews were conducted in isiXhosa, and required translation, thus creating the risk of nuances of meaning being lost during this process. The research team did not have the opportunity to validate the translations of collected data with participants. Finally, as interviews were conducted in the health-care facility where PNCs took place, participants may have been biased by their surroundings and emphasised advantages of the PNC model rather than discussing any challenges they had experienced.

## Conclusion

The PNC model triggered the transmission of knowledge about HIV, ART, infant feeding and early childhood development. Participation in PNCs resulted in behaviour change related to health and motherhood. Above all, by inviting postpartum women living with HIV to meet and share their concerns in groups, the PNCs generated peer support among members. Participants, staff and the majority of key informants expressed a high level of satisfaction with the PNC model. In addition to these benefits, the PNC model carries the advantage of combining PMTCT and MCH care.

One of the main challenges raised about the model was its sustainability in relation to workload and staffing issues. Lessons learned from other community models of care could provide hints on ways to ensure sustainability of the PNCs: some tasks, such as counselling, drug dispensation and administrative tasks, may be delegated to PNC graduates or PNC members.

## Data Availability

All the available collected data were included in the study. The datasets used and analysed during the current study are available from the corresponding author on reasonable request.

## Abbreviations

ART: Antiretroviral Treatment
CAG: Community ART Group
ECD: Early Childhood Development
FGD: Focus Group Discussion
HIV: Human Immunodeficiency Virus
IDI: In-Depth Interview
m2m: mothers2mothers
MCH: Mother and Child Health
MSF: Médecins Sans Frontières
MTCT: Mother To Child Transmission
PMTCT: Prevention of Mother to Child Transmission
PNC: Post Natal Club
PI: Principal investigator
PLWHIV: People Living With HIV
